# Temperature-Dependent Electrochemical Stability Window of Bis(trifluoromethanesulfonyl)imide and Bis(fluorosulfonyl)imide Anion Based Ionic Liquids

**DOI:** 10.3389/fchem.2022.859304

**Published:** 2022-06-17

**Authors:** Kallidanthiyil Chellappan Lethesh, Ahmed Bahaa, Mariam Abdullah, Musbaudeen O. Bamgbopa, Rahmat Agung Susantyoko

**Affiliations:** Research and Development Centre, Dubai Electricity and Water Authority (DEWA), Dubai, United Arab Emirates

**Keywords:** electrochemical stability, temperature, linear regression, TFSI, FSI, bis(trifluoromethanesulfonyl)imide, bis(fluorosulfonyl)imide, ionic liquids

## Abstract

The electrochemical stability of 22 commercially available hydrophobic ionic liquids was measured at different temperatures (288.15, 298.15, 313.15, 333.15 and 358.15 K), to systematically investigate ionic liquids towards electrolytes for supercapacitors in harsh weather conditions. Bis(trifluoromethanesulfonyl)imide and bis(fluorosulfonyl)imide anions in combination with 1-Butyl-1-methylpyrrolidinium, 1-Ethyl-3-methylimidazolium, N-Ethyl-N, N-dimethyl-N(2methoxyethyl)ammonium, 1-Methyl-1-(2-methoxyethyl)pyrrolidinium, N-Pentyl-N-methylpyrrolidinium, N, N-Diethyl-N-methyl-N-propylammonium, N, N-Dimethyl-N-ethyl-N-benzyl ammonium, N, N-Dimethyl-N-Ethyl-N-phenylethylammonium, N-Butyl-N-methylpiperidinium, 1-Methyl-1-propylpiperidinium, N-Tributyl-N-methylammonium, N-Trimethyl-N-butylammonium, N-Trimethyl-N-butylammonium, N-Trimethyl-N-propylammonium, N-Propyl-N-methylpyrrolidinium cations were selected for the study. Linear regression with a numerical model was used in combination with voltammetry experiments to deduce the temperature sensitivity of both anodic and cathodic potential limits (defining the electrochemical stability window), in addition to extrapolating results to 283.15 and 363.15 K. We evaluated the influence of the cations, anions, and the presence of functional groups on the observed electrochemical stability window which ranged from 4.1 to 6.1 V.

## 1 Introduction

Ionic liquids (ILs) are being used to replace conventional organic solvents in various applications because of their unique features such as inherent ionic conductivity, high thermal stability, wide liquid state temperature range, and high electrochemical stability (Wasserscheid and Welton, 2008) (Paul et al., 2020) (Lethesh et al., 2021) (Kermanioryani et al., 2016) (Grøssereid et al., 2019). Recently, the application of ionic liquid (IL) based electrolytes in energy storage devices has been an active area of research (Chellappan et al., 2020) (Bahadori et al., 2020) (Doherty, 2018) (Ma et al., 2018). The systematic measurement and analysis of IL electrochemical stability window ( ESW ) is essential in developing IL based electrochemical systems. The ESW is the electric potential window bounded at both its positive and negative limits by anodic and cathodic IL degradation potentials ( $E_{a}$ and $E_{c}$ ), respectively, and can be defined by Eq. 1. Given the chemical structure of a typical IL is that of a paired anion and cation, the $E_{a}$ is attributed to the oxidation potential of the ILs constituent anion, while $E_{c}$ is attributed to the reduction potential of the constituent cation (Zhang et al., 2006). At potentials beyond the ESW , electrochemical systems like batteries and supercapacitors applying ILs become unstable because of degradation of the ILs, due to the undesired reactions which occur around $E_{a}$ and $E_{c}$ .

$$ESW=E_{a}-E_{c}$$ (1)

As observed experimentally, recorded faradaic currents describing $E_{a}$ and $E_{c}$ —when measured with potential sweep voltammetry are affected by different factors. Some factors are: the type and morphology/structure of the working electrode (Xue et al., 2018) (Cao et al., 2012) and impurities. Impurities in the ILs can detrimentally affect the electrochemical stability (subsequently ESW ) of ILs. Therefore, rigorous post-synthesis purification procedures are often required before applying ILs in electrochemical systems. For example, halide ion impurities present in the ILs (like ${\text{Cl}}^{-}$ , ${\text{Br}}^{-}$ , ${\text{I}}^{-}$ ) can get adsorbed on the electrode surface, which will reduce the surface fraction available for desired electrochemical reactions. The easily oxidisable nature of the halide ions can reduce the anodic limit ( $E_{a}$ ) of the ILs (Buzzeo et al., 2004).

The presence of water also reduces the electrochemical stability of ILs due to hydrogen and oxygen evolution reactions from the electrolysis of water (O’Mahony et al., 2008). In addition, the combination of I^−^ and water can form electroactive species, while HF can also be formed by the reaction of [PF_6_]^−^/[BF_4_]^−^ anions with water (Huddleston et al., 2001). The purification of ILs is energy extensive and can be quite complex, making it impractical for large-scale applications. Therefore, it is crucial to evaluate the electrochemical properties of commercial ILs to be used in large scale electrochemical applications like supercapacitor development (Lethesh et al., 2021).

This work focuses on ESW measurement and related analyses of the electrochemical stability of 22 commercially available bis(trifluoromethanesulfonyl)imide and bis(fluorosulfonyl)imide anion-based ILs between 283.15 and 363.15 K. These ILs are selected because of their hydrophobic nature and high thermal stability–making them promising candidates at higher temperatures. In addition, they are non-reactive with water and have simple synthesis and purification process (Wasserscheid and Welton, 2008) (Cao and Mu, 2014) (Freire et al., 2007). The motivation of the present study is to screen promising commercial ILs towards high voltage supercapacitors and similar electrochemical energy storage systems for harsh outdoor weather conditions. Currently, there is limited focus of literature towards commercial electrochemical energy storage systems in such applications. Conventional applied organic-solvent based electrolytes, suffer from high internal resistance, high toxicity, high flammable electrolytes and low cyclability at elevated temperature (Lin et al., 2016) (Shen et al., 2021) (Zhang et al., 2016). In other words, lack of stable electrolytes is a major roadblock in developing electrochemical energy storage devices for these outdoor conditions (Lin et al., 2016). Evaluating the electrochemical stability of commercially available ILs at high temperatures could expedite the development of safer high-temperature electrolytes for EES systems and other commercial electrochemical processes. As an additional motivation, there has been no systematic study on the effect of temperature on the electrochemical stability of ILs, to the best of our knowledge.

## 2 Materials and Methods

### 2.1 Chemicals

The ILs used in this study are; [Pyr _1,4_][FSI], [Pyr _1,4_][TFSI], [Pyr _1,3_][FSI], [Pyr _1,3_][TFSI], [Pyr _1,102_][FSI], [Pyr _1,102_][TFSI], [Pyr _1,5_][TFSI], [Pyr _1,103_][TFSI], [Pip _1,3_][TFSI], [Pip _1,3_][FSI], [Pip _1,4_][TFSI], [EMIm][FSI], [EMIm][TFSI], [N _1,1, 2, 102_][FSI], [N _1,1, 2, 102_][TFSI], [N _1,1,2_,Benz][TFSI], [N _1,1,2_, PhenylEth][TFSI], [N _2,2,1, 102_][FSI], [N _4,4,4,1_][TFSI], [N _1,1,1,4_][TFSI], [N _1,1,1,6_][TFSI], [N _1,1,1,3_][FSI]. All ILs were purchased from *Solvionic* and used without further purification. The full names of the ILs, purity and further details are shown in Supplementary Table S1 of supporting information (SI). As per the material data sheet, the water content in all the ILs used were less than 20 ppm. Ferrocene (99%) was purchased from *Alfa Aesar*. All chemicals were handled in an Argon-filled glovebox (*Mbraun*), while the closed, completely secluded, air/water-free cell assemblies were transferred outside the glovebox for electrochemical testing.

### 2.2 Electrochemical Characterisation

Cyclic voltammetry (CV) was performed using a microcell setup (TSC 70 closed-cell, *rhd instruments*) with a potentiostat (AutoLab PGSTAT302N, *Metrohm*) and workstation. The microcell’s airtight compartment is insulated from the atmosphere and utilised in normal room conditions. The microcell setup establishes; a Pt crucible as a counter electrode and four separately connectable Pt wire ends as working electrodes. The exposed working electrode diameter to the IL electrolytes is 0.25 mm. An AgCl coated Ag wire in direct contact with the ILs being measured was used as a quasi-reference for all measurements. For each test, ca. 100 µL of IL was used in the microcell. The microcell setup can be seen in Supplementary Figure S1 of the supporting information (SI).

CV scans were done at a scan rate of 50 mV s⁻^1^ for all experiments within high enough vertex potentials to accommodate significant anodic and cathodic degradation currents. The microcell setup uses a Peltier element in the cell stand for active heating/cooling of the compartment for accurate temperature control from the potentiostat software. We further verified the temperatures on the cell stand to be consistent with set points with a thermal camera. The CV scans were performed at 288.15, 298.15, 313.15, 333.15 and 358.15 K in this study at standard pressure. The experiments were done twice for each IL-temperature point combination, while the last of 4 CV cycles for each test was selected for analyses–allowing the recording of steady-state voltametric responses.

To determine the values of the electrochemical stability limits $E_{a}$ and $E_{c}$ , a practical numerically consistent method described by Mousavi et al. (Mousavi et al., 2015) was used. In the method, both stability limits are estimated as potentials at the intersection of two tangents, one crossing the non-faradaic plateau of the CV and the other through anodic/cathodic faradaic current rise towards vertex potentials. A graphical description of the method on a sample CV from our experiments can be seen in Supplementary Figure S2 of the SI. This method was selected over the other popularly deployed peak-current cut-off ( $J_{cut-off}$ ) method. In general, the $J_{cut-off}$ applies an arbitrarily set current density limit for cathodic and anodic decomposition current rise for determining both $E_{c}$ and $E_{a}$ . Consequently, this $J_{cut-off}$ approach has various disadvantages (Xu et al., 2001) (Ue et al., 1994). The arbitrary selection of the $J_{cut-off}$ created different standards in the literature, which resulted in a considerable difference (>0.9 V) in the electrochemical stability of the same electrolytes system (DeVos et al., 2014). Such discrepancies make it difficult to compare some earlier reported data and eliminates the possibility of a correlation between the electrolyte structure and its electrochemical stability. In addition, mass transport has a significant influence on the electrochemical stability window determined by the $J_{cut-off}$ method, as the change in the concentration of electrolytes can also significantly affect the recorded electrochemical stability (Olson and Bühlmann, 2013).

Calibration of the quasi-reference was done to determine its formal potential and temperature coefficient vs. a known ferrocene/ferrocenium (Fc/Fc^+^) reference, as described by Bard and Faulkner (de Rooij, 2003). In summary, the calibration was done by determining the equilibrium potential of Fc/Fc^+^ redox couple in one of the ILs used ([EMim][TFSI]) at the tested temperatures (288.15, 298.15, 313.15, 333.15 and 358.15 K), using a small amount of ferrocene (0.1 mM) dissolved in the IL. The CV measurements for the calibration exercise (see Supplementary Figure S3 of the SI) shows the Ag/Ag^+^ quasi-reference is -0.355 V vs. Fc/Fc^+^ at standard temperature (298.15 K), with a temperature coefficient of about 0.65 mV/K. Given this magnitude of the estimated possible drift within our measured temperature range (only ∼0.046 V within the 70 K difference), all equilibrium potentials for $E_{a}$ , $E_{c}$ and subsequently obtained ESW are reported to only one decimal place (in V) for high confidence. In addition, there is no practical significance in reporting ESW variations in the order of tens on millivolts in applications like batteries and supercapacitors.

### 2.3 Linear Regression

Given that the CVs were performed at 288.15, 298.15, 313.15, 333.15 and 358.15 K. A linear regression model was used to fit the experimental $E_{a}$ and $E_{c}$ values and extrapolate to temperature values from 283.15 to 363.15 K. The single-variable linear regression model is similar to the Nernst equation derived potential difference when temperature deviates from standard temperature. The model described mathematically is Eqs 2, 3. In both equations, E is the predicted potential (for $E_{a}$ or $E_{c}$ ), T and $\tilde{T}$ are measured temperature and normalised temperature values respectively (in K). Although W and b is the slope and constant in the linear regression model respectively, both depict a temperature coefficient and the standard equilibrium potentials of $E_{a}$ or $E_{c}$ for the ILs, respectively.

$$E=W.\tilde{T}+b$$ (2)

$$\tilde{T}=T-298.15$$ (3)

Obtained values of W and b for each IL will be presented and discussed in subsequent sections of this work.

## 3 Results and Discussion

**FIGURE 1:**
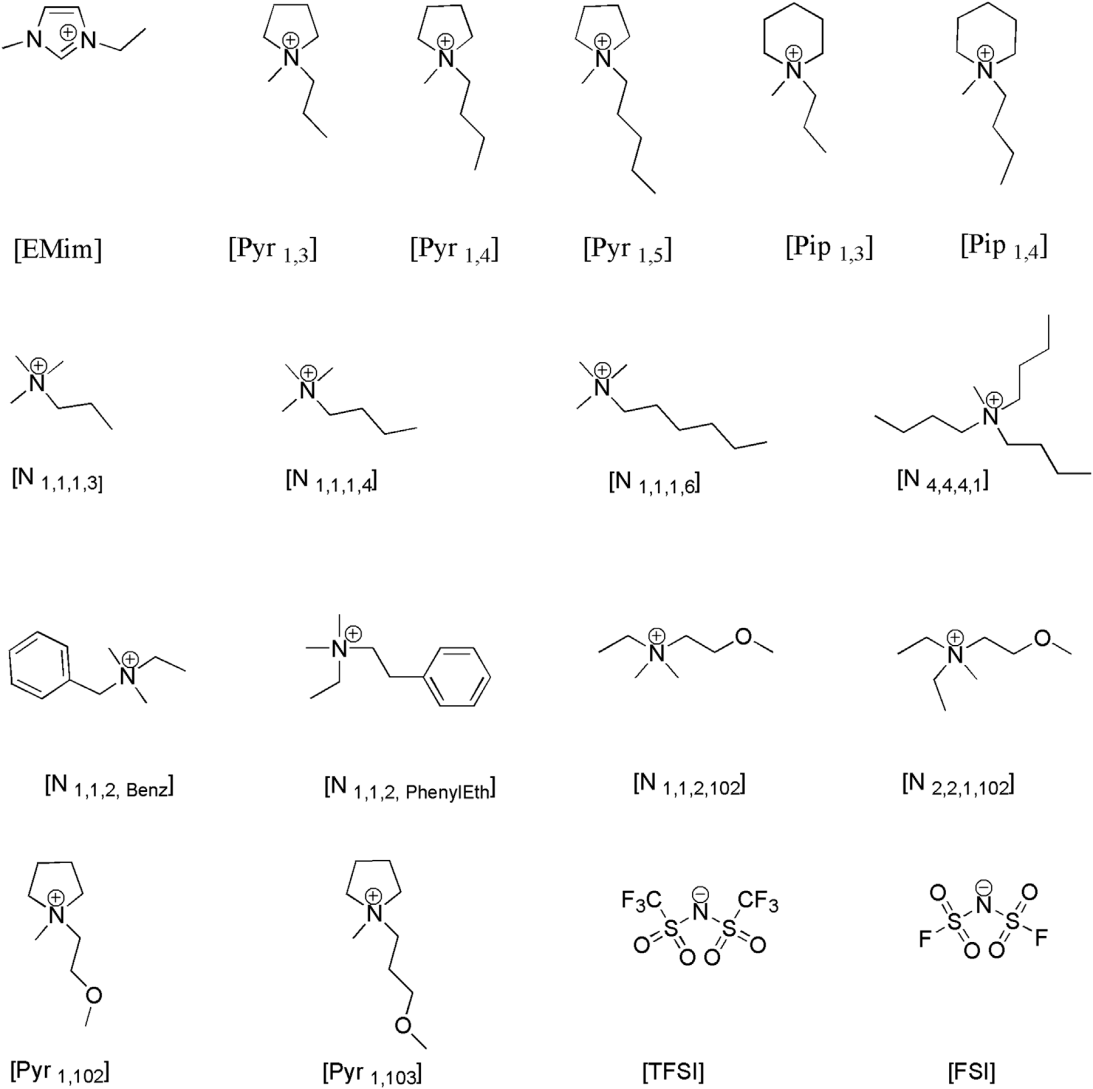
Structure of cations: 1-Ethyl-3-methylimidazolium, 1-Propyl-1-methylpyrrolidinium, 1-Butyl-1-methylpyrrolidinium, 1-Pentyl-1-methylpyrrolidinium, 1-Methyl-1-propylpiperidinium, N-Trimethyl-N-propylammonium, N-Trimethyl-N-butylammonium, N-Trimethyl-N-hexylammonium, N-Tributyl-N-methylammonium, N,N-Dimethyl-N-ethyl-N-benzylAmmonium, N,N-Dimethyl-N-Ethyl-N-Phenyl ethylammonium, N-ethyl-N,N-dimethyl-N-(2methoxyethyl)ammonium, N-N-Diethyl-N-methyl-N-(2methoxyethyl)ammonium,1-Methyl-1-(2-methoxyethyl)pyrrolidinium, 1-Methyl-1-(2-methoxypropyl)pyrrolidinium, and anions: bis(trifluoromethanesulfonyl)imide, bis(fluorosulfonyl)imide, under study.

Before presenting experimental results from the measurements, the structures of the ions making up the ILs used in this study are shown in Figure 1. In the figure, the chemical structure of the several cations paired with either bis(trifluoromethanesulfonyl)imide (TFSI) or bis(fluorosulfonyl)imide (FSI) anions are shown. The different Imidazolium, pyrrolidinium and piperidinium cations in combination with bis(trifluoromethanesulfonyl)imide ([TFSI]^-^) and bis(fluorosulfonyl)imide ([FSI]^-^) anions were selected because of their hydrophobicity.

### 3.1 Temperature-Dependent Electrochemical Stability Window

**FIGURE 2:**
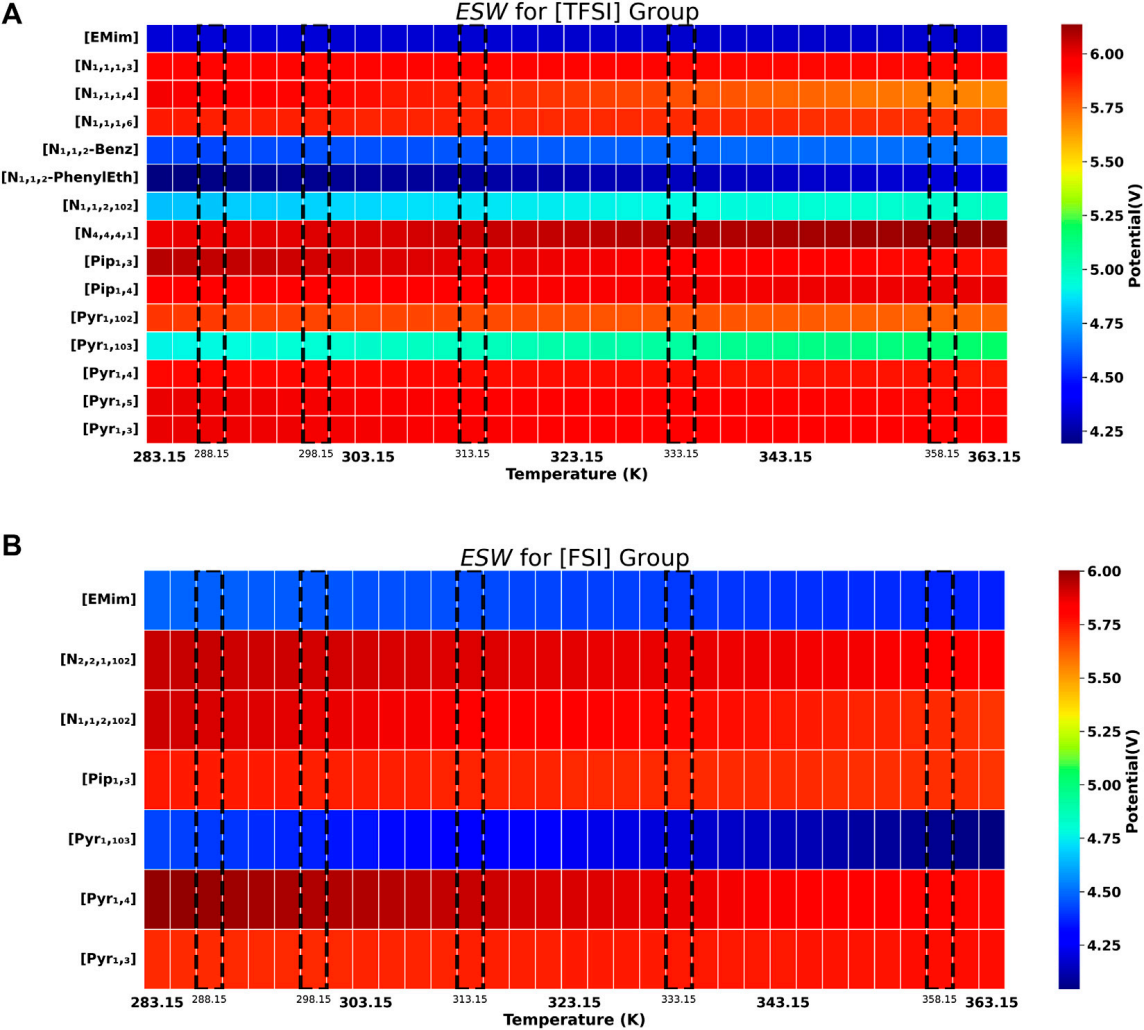
Electrochemical stability ( ESW ) of **(A)** [TFSI] anion group and **(B)** [FSI] anion group of ILs. Experimental values highlighted with dashed line frames and special ticks on the temperature axis.

Table 1 presents the anodic and cathodic potential limits ( $E_{\text{a}}$ and $E_{\text{c}}$ ) and obtained ESW from our experiments for all ILs at 288.15, 298.15, 313.15, 333.15 and 358.15 K. Figure 2A and Figure 2B also show the ESW graphically–using the regression model (described in Section 2.3) together with the experimental data, to extrapolate from 283.15 to 363.15 K for the ILs. The CV data from all tests of the ILs at the different temperatures are included in SI (Supplementary Figures S4, S5 and S6)—to avoid cluttering herein. An overview of Table 1 and both Figure 2A,B shows that 15 out of 22 ILs have ESW greater than 5.5 V and three ILs; [Pyr _1,3_][TFSI], [Pyr _1,5_][TFSI] and [N _4,4,4,1_][TFSI] have ESW of 6 V at 288.15 K.

**TABLE 1:** Measured $E_{a}$ and $E_{c}$ values of studied ionic liquids vs. Ag/Ag^+^, and obtained ESW .

Entry	ILs	288.15 K			298.15 K			313.15 K			333.15 K			358.15 K		
		*E*_c_	*E*_a_	*ESW*	*E*_c_	*E*_a_	*ESW*	*E*_c_	*E*_a_	*ESW*	*E*_c_	*E*_a_	*ESW*	*E*_c_	*E*_a_	*ESW*
		(V)			(V)			(V)			(V)			(V)		
1	[EMim][TFSI]	−1.8	2.5	4.4	−1.8	2.6	4.3	−1.8	2.3	4.4	−1.8	2.5	4.3	−1.8	2.0	4.3
2	[EMim][FSI]	−2.2	2.3	4.5	−2.2	2.3	4.5	−2.1	2.3	4.4	−2.0	2.3	4.3	−2.0	2.3	4.3
3	[Pyr _1,3_][TFSI]	−3.1	2.9	6.0	−3.1	2.9	6.0	−3.2	2.7	5.9	−3.2	2.8	6.0	−3.1	2.8	5.9
4	[Pyr _1,3_][FSI]	−2.7	2.9	5.6	−2.8	3.0	5.8	−2.8	3.0	5.8	−3.1	2.7	5.7	−2.9	2.8	5.7
5	[Pyr _1,4_][TFSI]	−3.2	2.7	5.9	−3.0	2.9	5.9	−3.2	2.8	6.0	−3.2	2.7	5.9	−3.1	2.8	5.9
6	[Pyr _1,4_][FSI]	−3.1	2.9	6.0	−3.1	2.8	5.9	−3.1	2.8	5.9	−3.1	2.8	5.9	−3.1	2.8	5.9
7	[Pyr _1,5_][TFSI]	−3.2	2.7	5.9	−3.2	2.7	5.9	−3.2	2.8	6.0	−3.0	2.9	5.9	−3.0	2.9	5.9
8	[Pyr _1,102_][TFSI]	−3.1	2.7	5.8	−2.9	2.9	5.8	−3.1	2.7	5.8	−2.8	3.0	5.8	−3.1	2.7	5.8
9	[Pyr _1,103_][TFSI]	−2.4	2.5	4.9	−2.4	2.5	4.9	−2.6	2.5	5.1	−2.6	2.5	5.1	−2.6	2.5	5.1
10	[Pyr _1,103_][FSI]	−2.2	2.0	4.2	−2.3	2.0	4.3	−2.3	2.0	4.3	−2.1	2.0	4.1	−2.1	2.0	4.1
11	[Pip _1,4_][TFSI]	−3.2	2.8	6.0	−3.1	2.9	6.0	−3.2	2.8	6.0	−3.2	2.8	6.0	−3.1	2.8	5.9
12	[Pip _1,3_][TFSI]	−3.3	2.7	6.0	−3.3	2.7	6.0	−3.3	2.8	6.1	−3.2	3.0	6.2	−3.1	3.0	6.1
13	[Pip _1,3_][FSI]	−2.9	2.9	5.8	−3.1	2.6	5.7	−2.9	2.9	5.8	−2.9	2.9	5.8	−3.0	2.7	5.7
14	[N _1,1,1,3_][TFSI]	−3.1	2.8	5.9	−3.0	2.9	5.9	−3.0	2.9	5.9	−3.0	2.8	5.8	−2.8	3.0	5.8
15	[N _1,1,1,4_][TFSI]	−3.0	2.9	5.9	−3.0	2.7	5.7	−3.0	2.9	5.9	−2.9	2.9	5.8	−2.9	2.8	5.7
16	[N _1,1,1,6_][TFSI]	−3.1	2.7	5.8	−2.8	3.0	5.8	−3.2	2.7	5.9	−3.1	2.7	5.8	−2.9	2.8	5.7
17	[N _4,4,4,1_][TFSI]	−3.2	2.8	6.0	−3.2	2.7	5.9	−3.1	2.9	6.0	−3.2	2.8	6.0	−3.1	2.8	5.9
18	[N _1,1,2,102_][FSI]	−3.2	2.7	5.9	−3.2	2.7	5.9	−3.1	2.7	5.8	−3.1	2.7	5.8	−2.9	2.9	5.8
19	[N _1,1,2,102_][TFSI]	−2.1	2.7	4.8	−2.1	2.7	4.8	−2.1	2.7	4.8	−2.2	2.7	4.9	−2.2	2.7	4.9
20	[N _2,2,1,102_][FSI]	−3.0	2.9	5.9	−3.1	2.8	5.9	−3.0	2.9	5.9	−3.0	2.8	5.8	−3.1	2.7	5.8
21	[N_1,1,2_-Phenyl Eth][TFSI]	−2.2	2.0	4.2	−2.2	2.0	4.2	−2.2	2.0	4.2	−2.1	2.1	4.2	−2.1	2.0	4.1
22	[N _1,1,2_-Benz][TFSI]	−2.2	2.3	4.5	−2.3	2.3	4.6	−2.3	2.3	4.6	−2.3	2.4	4.7	−2.2	2.4	4.6

Given the absence of data showing ESW at different temperatures in previous studies, a comparison of some of our obtained ESW values with previously reported data near room temperature for relevant ILs was established in Table 2.

**TABLE 2:** Comparison of some measured electrochemical stability window ( ESW ) of studied ILs with previously reported data at 298.15 K.

Entry	ILs	Working electrode	Reference electrode	ESW (V)	References
1	[EMim][TFSI]	Pt	I_3_/I^−^Ag/Ag ^+^	4.5	Bonĥte et al. (1996)
		GC	Ag/Ag^+^	4.7	Li et al. (2016)
		Pt		4.3	This work
2	[Pyr _1,3_][TFSI]	Pt	Fc/Fc^+^	5.6	Yim et al. (2007)
		Pt	Fc/Fc^+^	5.8	Appetecchi et al. (2009)
		Pt	Ag/Ag ^+^	6.0	This work
3	[Pyr _1,4_][FSI]	Unspecified	Unspecified	5.7	*Solvionic* (Reiter et al., 2013a)
		Pt	Li metal	5.8	This work
		Pt	Ag/Ag ^+^	5.9	
4	[Pyr _1,4_][TFSI]	GC	Ag wire	5.5	Sun et al. (1998)
		GC	Ag/Ag ^+^	5.8	MacFarlane et al. (1999)
		Pt	Ag/Ag^+^	5.9	This work
5	[Pip _1,3_][TFSI]	Pt	Ag ^0^/AgCF_3_SO_3_	5.0	Montanino et al. (2011)
		Pt	Fc/Fc^+^	5.2	Yim et al. (2007)
		Pt	Ag/Ag^+^	6.0	This Work
6	[Pip _1,4_][TFSI]	Pt	Silver wire	6.5	Gancarz et al. (2021)
		Pt	Li wire	5.6	Le et al. (2012)
		Pt	Ag/Ag^+^	6.0	This work
7	[N _1,1,1,4_][TFSI]	GC	Fc/Fc^+^	5.9	Maton et al. (2012)
		GC	Fc/Fc^+^	5.8	Maton et al. (2012)
		Pt	Ag/Ag^+^	5.7	This work
8	[N _4,4,4,1_][TFSI]	GC	Ag/Ag^+^	5.5	Li et al. (2016)
		GC	Ag/Ag^+^	5.6	Xue et al. (2018)
		Pt	Ag/Ag^+^	5.8	This work
9	[N _1,1, 2, 102_][TFSI]	GC	Fc/Fc^+^	5.5	DeVos et al. (2014)
		GC	Ag/Ag^+^	5.9	Hayyan et al. (2013)
		Pt	Ag/Ag^+^	5.9	This work
10	[N _2,2,1,102_][TFSI]	GC	I_3_/I^−^Fc/Fc^+^	5.7	Zhou et al. (2005)
		Pt	Ag/Ag^+^	5.5	Kim et al. (2005)
		Pt		5.9	This work

It can be seen in the Table that the ESW obtained in our study (at room temperature) agrees with the literature values, especially on the similar working electrode material (Pt). For example, an ESW value of 4.3 V was obtained for [EMim][TFSI] in our study at 288.15 K, which is in good agreement with the 4.5 V reported in similar experimental conditions (Bonĥte et al., 1996). Noticeable differences in ESW values were observed between the literature reported values and results obtained in our study for some ILs (like [Pip _1,3_][TFSI], [Pip _1,4_][TFSI] and [Pyr _1,3_][TFSI], even with the same working electrode, which might be because of the difference in the purity of ILs used (Randström et al., 2008).

Interestingly, only one ILs, [Pip _1,3_][TFSI] showed ESW of more than 6 V (at 313.15 K). It had been reported that the increase in temperature generally decreases the ESW of ILs (Koch et al., 1996; Randström et al., 2008). Although we observe minor decrease in ESW with temperature (in order of hundred mV) for most ILs within the measured temperature range under study, our measurements also show different trends for some ILs. Some ILs had ESW remain the same ([Pyr _1,3_][FSI], [EMim][TFSI], [Pip _1,4_][TFSI]) or had minor increase ([Pip _1,3_][TFSI], [N _1,1,2_-Benz][TFSI]).

Theoretically, the voltametric response recorded with the CVs at different temperatures is governed by the interplay of thermodynamic, reaction kinetics and mass transport influences. Temperature influence on the thermodynamic equilibrium potential of reactions at both anodic and cathodic limits is well described with the Nernst equation. Increasing temperature is generally expected to favour reaction kinetics, as seen in Butler-Volmer-type kinetic relations (de Rooij, 2003). Furthermore, the sensitivity of IL viscosities (and their ionic conductivities/mobilities) to temperature varies significantly among the different IL groups, thereby affecting ionic transport to and from the electrode surface. All else being equal—if the ionic mobility/diffusivity for some ILs does not significantly increase within the reported temperature range, the resulting change in $E_{\text{a}}$ , $E_{\text{c}}$ and subsequently, ESW will be minimal. This trend was observed in ESW of ILs like [Pyr _1,102_][TFSI] from our experiments. Overall, in our measurements, significant changes in ESW for the ILs were only noticed approaching higher temperature beyond 358.15 K (see Figure 2A,B).

**FIGURE 3:**
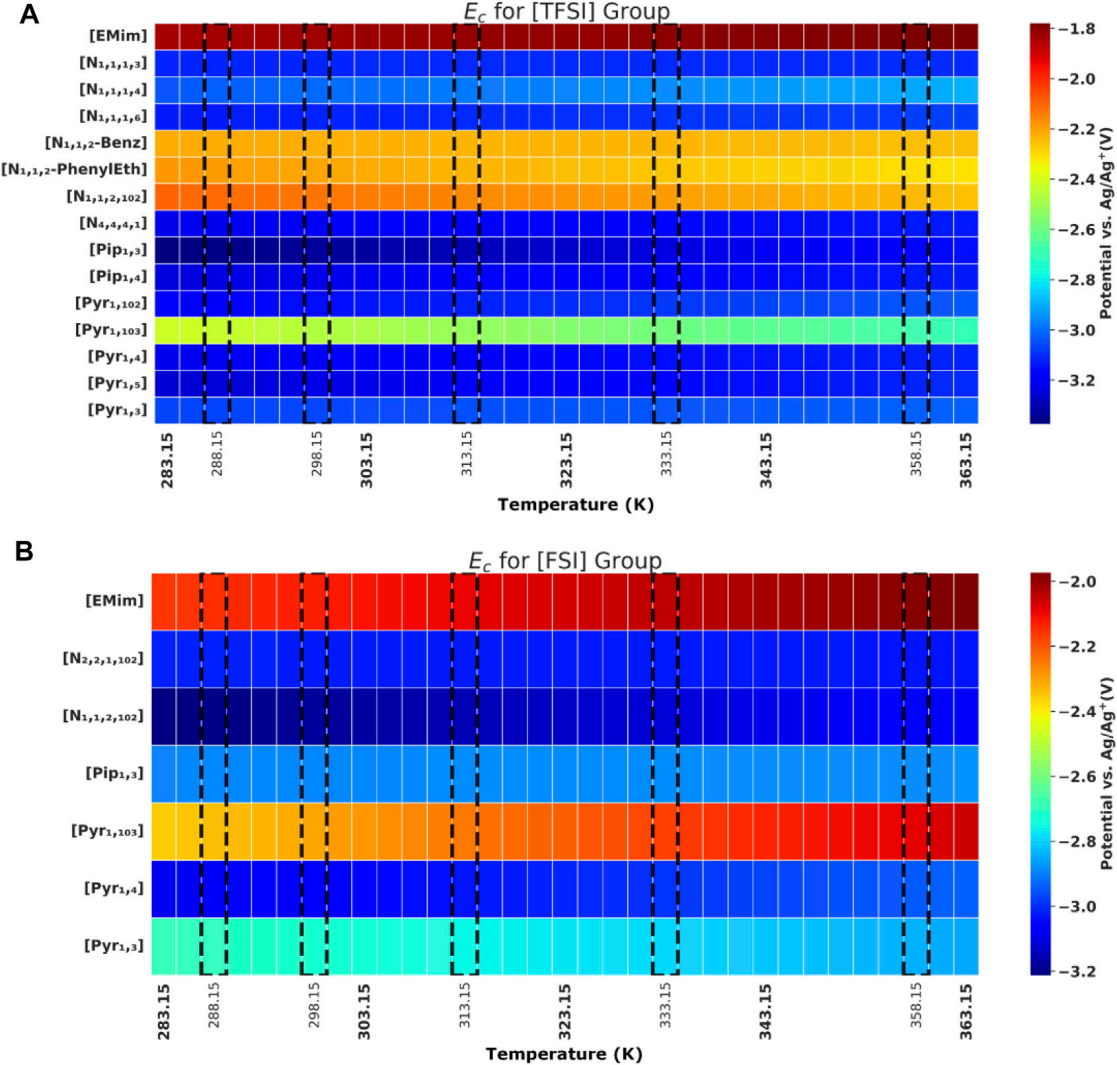
Cathodic potential ( $E_{c}$ ) values of ILs; **(A)** [TFSI] anion group. **(B)** [FSI] anion group. Experimental values highlighted with dashed line frames and special ticks on temperature axis.

**FIGURE 4:**
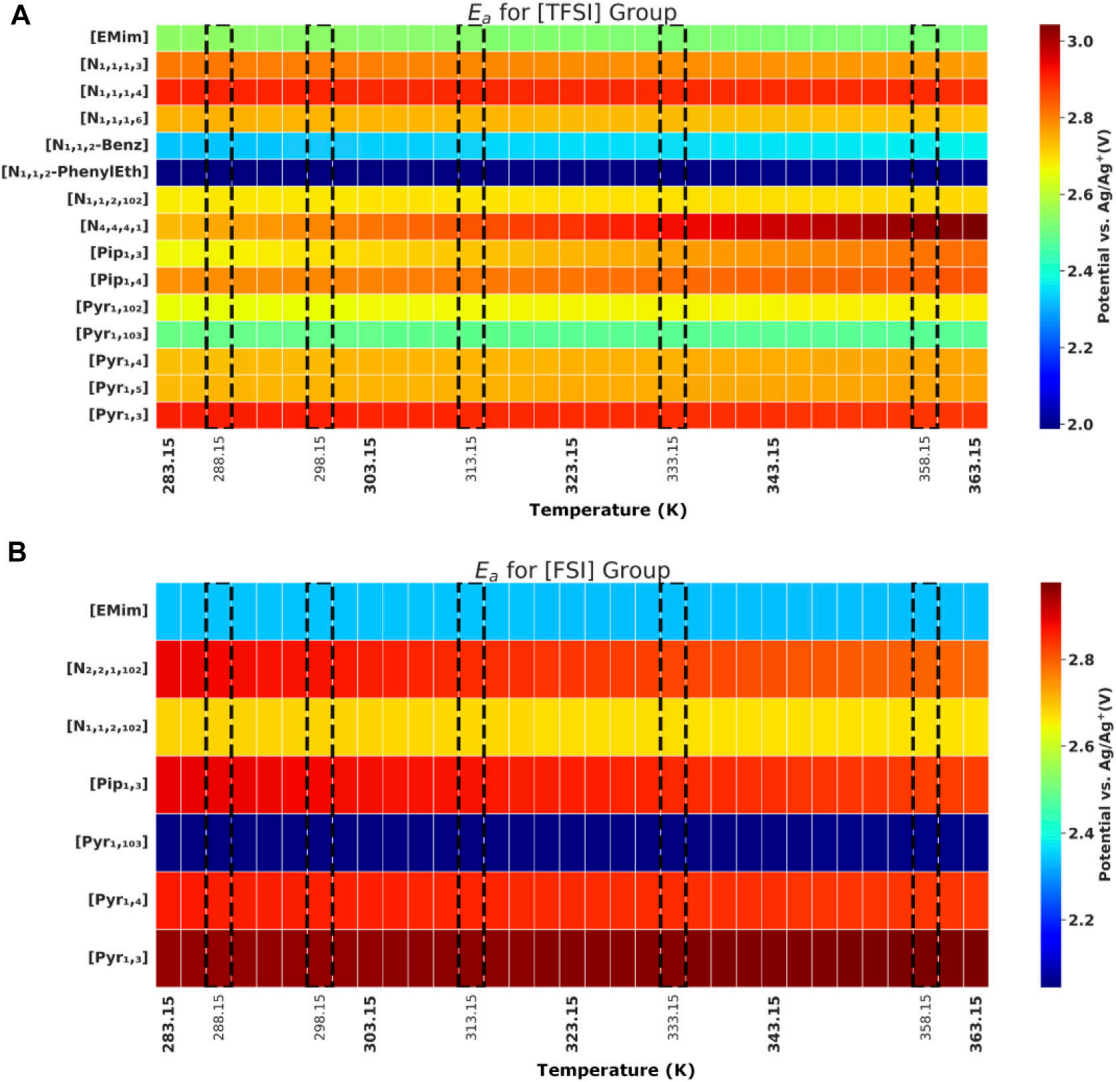
Anodic potential ( $E_{a}$ ) values of ILs; **(A)** [TFSI] anion group. **(B)** [FSI] anion group. Experimental values highlighted with dashed line frames and special ticks on the temperature axis.

As explained previously in Section 2.3, the slope and constant ( W and b , respectively in Eq. 2) represent the standard potential values and a temperature coefficient/sensitivity–when Eqs 2, 3 is written for $E_{\text{a}}$ or $E_{\text{c}}$ . This allows us to attempt to deduce the influence of cations and anions on $E_{\text{a}}$ and $E_{\text{c}}$ , respectively, and subsequently ESW from the ion pairs. Table 3 presents the results of W and b from the linear regression. Positive W value indicates an increase for $E_{\text{a}}$ or $E_{\text{c}}$ with increasing temperature, while negative W suggests a decrease of the values with increasing temperature (see Eqs 2, 3. The recorded $E_{c}$ at different temperatures are shown graphically in Figure 3A and Figure 3B, while the recorded $E_{a}$ at different temperatures are also shown graphically in Figure 4A,B.

**TABLE 3:** Learned model (Eq. 2) parameters of [TFSI] and [FSI] group ILs with the paired cations^a^.

IL cations	[TFSI] group				[FSI] group			
	$E_{a}$ (V)		$E_{c}$ (V)		$E_{a}$ (V)		$E_{c}$ (V)	
	W (mV/K)	b (V)	W (mV/K)	b (V)	W (mV/K)	b (V)	W (mV/K)	b (V)
[EMim]	−0.3	2.5	0.7	−2.0	−0.1	2.3	2.4	−2.8
[Pip_1,3_]	2.0	2.6	2.9	−4.2	−0.8	2.9	0.2	−2.9
[Pip_1,4_]	0.8	2.8	1.4	−3.7	-	-	-	-
[Pyr _1,102_]	0.3	2.7	1.9	−3.7	-	-	-	-
[Pyr _1,103_]	−0.3	2.5	−3.7	−1.4	0.1	2.0	3.8	−3.4
[Pyr _1,3_]	−0.4	2.9	0.6	−3.2	0.3	3.0	−2.1	−2.1
[Pyr _1,4_]	0.4	2.7	1.3	−3.6	−0.4	2.9	1.9	−3.6
[Pyr _1,5_]	0.4	2.7	2.0	−3.8	-	-	-	-
[N_1,1,2,102_]	0.2	2.7	−2.0	−1.5	−0.2	2.7	2.0	−3.8
[N _2,2,1,102_]	-	-	-	-	−1.3	2.9	−0.2	−3.0
[N _1,1,1,3_]	−0.5	2.8	0.2	−3.2	-	-	-	-
[N _1,1,1,4_]	−0.1	2.9	1.7	−3.5	-	-	-	-
[N _1,1,1,6_]	−0.3	2.8	0.8	−3.3	-	-	-	-
[N_1,1,2_-Benz]	0.6	2.3	−0.5	−2.1	-	-	-	-
[N_1,1,2_-PhenylEth]	0.2	2.0	−1.6	−1.7	-	-	-	-
[N_4,4,4,1_]	3.8	2.7	1.1	−3.5	-	-	-	-

a W and b are expressed accordingly for consistent comparison of magnitudes with values of potential obtained from experimental results. Dash (-) means not tested.

### 3.2 Effect of Cation

**SCHEME 1:**
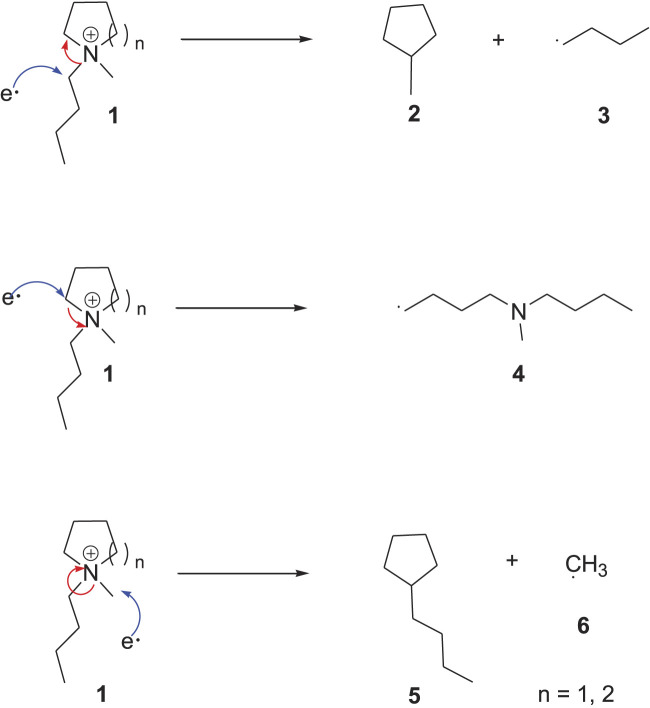
Electrochemical decomposition of pyrrolidinium/piperidinium cation (Kroon et al., 2006).

The structure of the ILs (cations and anions) greatly influences their electrochemical stability. Among different cations studied, piperidinium and pyrrolidinium cations show the highest ESW at all temperatures, followed by ammonium cations, which agrees with previous studies (Liu et al., 2010). The higher electrochemical stability of pyrrolidinium and piperidinium ILs attributed to their different electrochemical decomposition mechanisms than ammonium and imidazolium cations (Handy and Okello, 2005). The pyrrolidinium ring undergoes decomposition in totally different pathways than the imidazolium cation. The electrochemical decomposition of the pyrrolidinium/piperidinium cation might happen in three different ways, as seen in Scheme 1 (Kroon et al., 2006). The most probable decomposition path is the formation of the N-methyl pyrrolidine (**2**) because of the stability of the butyl radical (**3**). A less likely but viable route is the ring-opening of the cationic core to dibutyl methylamine radical **4**. The lowest possible way is the degradation of the cation into N-butyl pyrrolidine (**5**) and methyl radical **6** due to the instability of the methyl radical.

**SCHEME 2:**
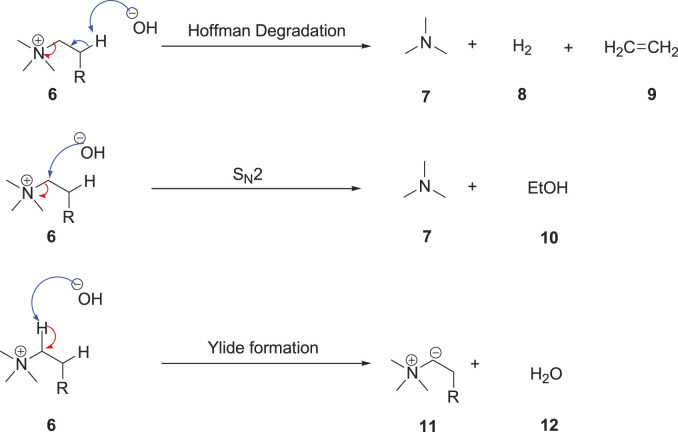
Degradation pathways of ammonium ILs (Ramnial et al., 2008).

It has been reported that ESW of ILs increases with the increase in the alkyl spacer length on the cation (Xue et al., 2018) (Hayyan et al., 2013). Table 3 shows standard $E_{c}$ ( b in the table) became more electronegative as alkyl spacer length on the pyrrolidinium cations increase (from [Pyr _1,3_]^+^ to [Pyr _1,4_]^+^ to [Pyr _1,5_]^+^), when they are paired with both [TFSI]^-^ and [FSI]^-^ anions. $E_{c}$ becoming more electronegative (i.e., reducing numerically) should normally indicate higher ESW if $E_{a}$ on the positive side was same. The trend in $E_{c}$ with pyrrolidinium cations might be due to the high electron-donating ability of the alkyl side chains with the increase in the number of carbon atoms, which shields the positively charged nitrogen atom on the pyrrolidinium cation from electrochemical reduction (Appetecchi et al., 2009). The literature values reported (Lian et al., 2019) (Hayyan et al., 2013) (Neale et al., 2017) (Zhang and Bond, 2005) for the $E_{c}$ values for the pyrrolidinium ILs is in good agreement with our study. For ammonium cations, mixed trend for $E_{c}$ is observed, with [N _1,1,1,3_]^+^, [N _1,1,1,4_]^+^, and [N _1,1,1,6_]^+^ when paired with [TFSI]^-^, which might be because of their difference in the decomposition mechanism. The ammonium ILs can undergo degradation in two different pathways: Hoffman degradation and bimolecular nucleophilic substitution (S_N_2) reaction (Scheme 2) (Ramnial et al., 2008). The most likely pathway is the Hoffman elimination, which mainly depends on the nature of the alkyl groups and substitution pattern on the ammonium cation (Long et al., 2012), which can create steric hindrance for the abstraction of proton from the β position of the nitrogen atoms (Long et al., 2012). In other words, the stability of ammonium ILs can be improved by increasing the alkyl spacer length or introducing substituents on the β position (Lethesh et al., 2014). Ammonium ILs can also undergo decomposition through the ylide formation by the abstraction of the α-hydrogen atom.

Table 3 also shows that the magnitude of $E_{c}$ sensitivity to temperature ( W in the table) is highest for [Pyr _1,103_]^+^ cation, at −3.7 and 3.8 mV/K, when paired with both [TFSI]^-^ and [FSI]^-^ respectively. [N _1,1,1,3_][TFSI], [Pip_1,3_][FSI] and [N _2,2,1,102_][FSI] have the lowest temperature sensitivity with, 0.2, 0.2, −0.2 mV/K, respectively.

The expected, resulting increase in ESW with the increasing alkyl spacer length on the cation can be related to the electron-donating nature of the alkyl groups, which will reduce the positive charge on the nitrogen and decrease its vulnerability towards electrochemical reduction (Taeeun et al., 2009). The earlier reported ESW values for the ammonium ILs (Xue et al., 2018) (Lian et al., 2019) are comparable to our results (see Table 2). The pyrrolidinium and piperidinium ILs had exceptional electrochemical stability because of their resistance towards reduction due to the absence of unsaturated (double bond/triple bond/aromatic) groups. Tetralkylammonium cations with longer alkyl groups showed almost similar ESW and $E_{c}$ values to the pyrrolidinium cations, which agrees with the earlier report in literature (Maton et al., 2013).

**SCHEME 3:**
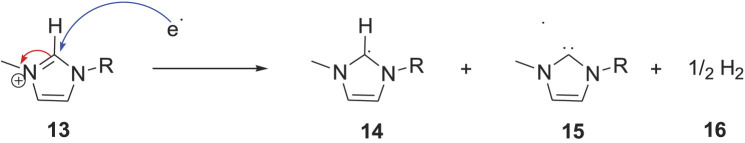
Imidazolium cation reduction by carbene and radical formation(Xiao, 2002) (Noack et al., 2010).

As expected, both Imidazolium ILs (with [EMim]^+^ cation) showed the lowest ESW and $E_{c}$ values at all temperatures under study because of their vulnerability towards electrochemical reduction due to the presence of proton on the C2 position on the imidazolium ring (Bonĥte et al., 1996). As shown from Scheme 3, the C2 hydrogen is responsible for the electrochemical reduction of the imidazolium ring (Xiao, 2002) (Noack et al., 2010).

**SCHEME 4:**
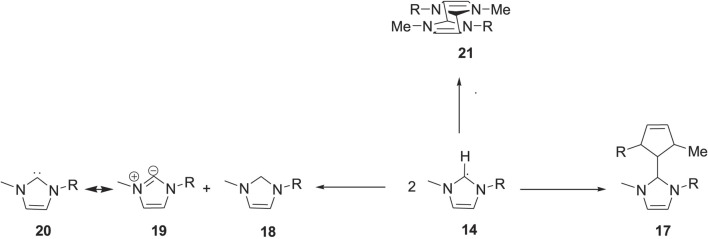
Dimer and cage-like structure formation of the imidazolium (Xiao, 2002).

Nuclear magnetic resonance (NMR) spectroscopy analysis of the imidazolium ILs after continuous electrolysis showed that the C2 position of the imidazolium ring was altered and resulted in the loss of aromaticity of the imidazolium cation and the formation of a neutral radical **14**. The radical can subsequently convert to carbene **15** with the evolution of H_2_ gas (Noack et al., 2010). This carbene can exist in the ILs phase if the substituents on the imidazolium nitrogen atoms are suitable. Otherwise, it can react further and result in the formation of dimer **17,** or it can react with other imidazolium species and form a saturated C2 carbon atom containing cage-like molecule **21,** as shown in Scheme 4 (Xiao, 2002).

The C2 hydrogen is also primarily responsible for the thermal degradation of imidazolium ILs. The C2 hydrogen is acidic and can undergo deprotonation even in neutral media if the anion is slightly basic (Handy and Okello, 2005). In the presence of strongly basic anions such as hydroxide (Noack et al., 2010), the deprotonation occurs faster. It will result in the formation of N-heterocyclic carbenes (Díez-González et al., 2009), which eventually opens the imidazolium ring by the nucleophilic addition of the hydroxide ion (Chetsanga and Makaroff, 1982).

The $E_{c}$ values of Imidazolium ILs increased when the [TFSI]^-^ anion was replaced with [FSI]^-^ anion. In contrast, a significant difference in $E_{c}$ values were observed in the case of [Pip _1, 3_][TFSI] (-3.3 V) and [Pip _1, 3_][FSI] (-2.9 V).

### 3.3 Effect of Anions

**SCHEME 5:**
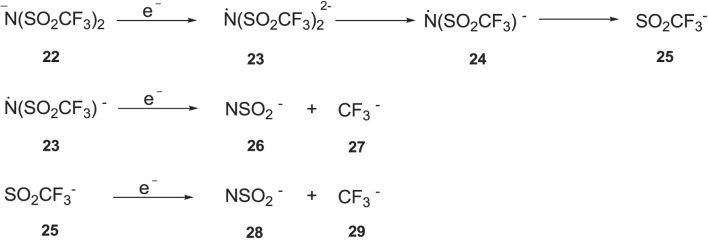
Electrochemical degradation of [TFSI] anion (Howlett et al., 2006).

It has been reported (Matsumoto et al., 2005) that [TFSI]^-^ anion based ILs have wider ESW compared to other anions because of their ability to delocalize the negative charge on the nitrogen atom on the entire molecule. However, studies revealed that two electrons withdrawing groups (S=O) reduce the partial charges on the nitrogen atom, which make them susceptible to reduction. In addition, the lowest unoccupied molecular orbital (LUMO) of the reduced species situated on the sulphur atom and addition of the electrons occurs there, which will weaken the S-N bond and lead to the decomposition of the [TFSI]^-^ anion into nitrogen centred radical as can be seen from (Scheme 5) (Howlett et al., 2006) (Randström et al., 2008)^,^ (Randström et al., 2007). The radical (**24**) and the small anion (**25**) formed undergo further decomposition into smaller anions (**26**, **27** and **28**).

Comparing ILs herein with both [TFSI]^-^ and [FSI]^-^ anions, we observed a mixed trend in electrochemical stability (Table 3 and Figure 4), which is largely due to the similarity in both anion structures. In the case of Imidazolium ILs, the [TFSI]^-^ anions have a noticeable higher $E_{a}$ values (0.3 V) compared to their [FSI]^-^ analogue, while the ESW remains the same for both ILs, which is not surprising because earlier studies revealed that the oxidation and reduction of imidazolium cation were not dependent on the anions used (Matsumoto et al., 2005).

In general, ILs with [FSI] anion showed higher Ec in comparison to its [TFSI] anion counterpart. Although the cathodic limit is largely attributed to the reduction potential of the constituent cation (earlier hinted in introduction), earlier discussions (DeVos et al., 2014) also indicate that for some ion pairs, further reduction of the anion could be preferential at the cathode–just as further oxidation of the cation could also be preferential on the anode. This could explain out observations why; out of 6 cations combined with both [TFSI] and [FSI] in our study, three of them showed this trend (Ec higher with FSI than TFSI), two showed the opposite, while only one reported same value for Ec (when [TFSI] and [FSI] are in the presence of [Pyr _1,4_] cation). The actual Ec values tagged when [FSI] and [TFSI] are paired with the same cation could also differ given ion mobility variation with different bond strength with specific cation, among other reasons.

For ether functionalized ILs (like [N _1,1,2,102_][TFSI] and [N _1,1,2,102_][FSI]), $E_{a}$ values remained the same (2.7 V) for both [TFSI]^-^ and [FSI]^-^ anions, while a significant difference in the ESW was observed between [FSI]^-^ (5.8 V) and [TFSI]^-^ (4.9 V) anion pairs.

Although [TFSI]^-^ anion paired with pyrrolidinium ILs showed lower $E_{a}$ values (2.8–2.9 V) than [FSI]^-^ anion (2.9–3.0 V) based ones, the ESW was slightly higher with the former anions.

The ESW values for pyrrolidinium ILs obtained in our study is comparable with the literature reported data (5.2–5.8 V) (Neale et al., 2016) (Randström et al., 2008) (Reiter et al., 2013b). A noticeable difference in $E_{a}$ values was observed for ether functionalized pyrrolidinium ILs when the [TFSI]^-^ anion was replaced with [FSI]^-^. For piperidinium ILs, $E_{a}$ values increased by 0.2 V when the anion changed from [TFSI]^-^ to [FSI]^-^, which is in accordance with the literature data (Matsumoto et al., 2005).

For $E_{a}$ sensitivity to temperature, the recorded values were generally less than or around 1 mV/K ( W in Table 3), except for [N _4,4,4,1_][TFSI] and [Pip_1,3_][TFSI], with 3.8 and 2.0 mV/K, respectively).

### 3.4 Effect of Functional Groups

In general, the inclusion of functional groups can affect the properties of ILs. For example, the introduction of ether groups on IL cations can reduce their viscosity (Raj et al., 2019). It was suspected that the ether functionality could increase the $E_{c}$ of ILs due to the interaction between lone pair of electrons on the ether oxygen and a positively charged nitrogen atom, which can shield cation from the electrochemical reduction. In contrast, we observed that the $E_{c}$ was reduced because of the decrease in the electron density of the ether oxygen atom due to the interaction. Another reason behind the lowering of $E_{c}$ of ether functionalised ILs is their weaker ability to donate electrons to the positively charged nitrogen atom than simple alkyl groups (Pandian et al., 2020). Similar results were observed in our study. As can be seen from Table 1, replacement of methoxyethyl group ([Pyr _1,102_][TFSI]) with methoxy propyl group ([Pyr _1,103_][TFSI]) reduced the standard (room temperature) ESW from 5.8 to 5.0 V.

The introduction of the aromatic groups on ammonium cations has a similar effect as the ether functionality. The presence of the aromatic groups reduced the electrochemical window considerably. The standard ESW of ammonium ILs decreased when phenylethyl and benzyl groups were attached to the ammonium cations instead of the alkyl groups (4.3 and 4.5 V, for phenylethyl and benzyl groups, respectively). This reduction in the ESW value might be related to the quickly reducing nature of the aromatic groups (Lethesh et al., 2019). It is worth mentioning that the temperature has only a negligible influence on the $E_{c}$ values of ether functionalised ILs.

## 4 Conclusion

Twenty two commercially available ionic liquids with bis(trifluoromethanesulfonyl)imide and bis(fluorosulfonyl)imide anions were used to investigate temperature effect on electrochemical stability window on ionic liquids. Within the temperature range investigated (283.15–363.15 K), the increasing temperature had mixed results on the electrochemical stability of ionic liquids with different cation types investigated. Although both anions used in the study have a similar structure, bis(trifluoromethanesulfonyl) imide anions showed slightly higher electrochemical stability overall than bis(fluorosulfonyl)imide anions.

We confirm that increase in the alkyl spacer length on the cation increased ionic liquids’ electrochemical stability compared to their short alkyl chain counterpart. In contrast, the aromatic functional groups on the cation significantly reduced their electrochemical stability window, as seen in the case of N, N-Dimethyl-N-ethyl-N-benzylammonium bis(trifluoromethanesulfonyl)imide. The presence of ether functionality on the cationic core also reduces the electrochemical stability window, which is evident in the case of N-Ethyl-N, N-dimethyl-N-(2-methoxyethyl) ammonium bis(trifluoromethanesulfonyl)imide. Among the different ionic liquids studied, Imidazolium ionic liquids (1-Ethyl-3-methylimidazolium bis(fluorosulfonyl)imide) showed the lowest electrochemical window and pyrrolidinium (N-Pentyl-N-methylpyrrolidinium bis(trifluoromethanesulfonyl)imide) and tetraalkylammonium ionic liquids (N-Tributyl-N-methylammonium bis(trifluoromethanesulfonyl)imide) with more extended alkyl groups showed the largest electrochemical stability window.

The limited variation of ionic mobilities/diffusivities within the temperature range investigated is a significant factor behind generally observed changes for anodic, cathodic, and subsequently electrochemical stability of the ILs. Given the different cation types were paired with two anion types, recorded cathodic potential limits were more sensitive to increasing temperature than the anodic potential limits.

## Data Availability

The raw data supporting the conclusion of this article will be made available by the authors, following institutional clearance.
