# Supplementary material for: Temperature-Dependent Electrochemical Stability Window of Bis(trifluoromethanesulfonyl)imide and Bis(fluorosulfonyl)imide Anion Based Ionic Liquids
Source: Front Chem. 2022 Jun 17;10:859304. doi: 10.3389/fchem.2022.859304 (PMC9247390; doi:10.3389/fchem.2022.859304)
Supplement: Supplementary file 1 [file DataSheet1.pdf]

Supporting information

Temperature-dependent electrochemical stability window of bis(trifluoromethanesulfonyl)imide and bis(fluorosulfonyl)imide anion based ionic liquids

Kallidanthiyil Chellappan Lethesh[1], Ahmed Bahaa[1], Mariam Abdullah, Musbaudeen O. Bamgbopa, Rahmat Agung Susantyoko*

Research and Development Centre, Dubai Electricity and Water Authority (DEWA), Dubai, United Arab Emirates.

[1]both authors contributed equally

*Corresponding author

Dr. Rahmat Agung Susantyoko

Email: rahmat.susantyoko@dewa.gov.ae , rahmat.a.susantyoko@alum.mit.edu

Table S1: Name and specifications of ILs used

Entry	ILs	Abbreviation	CAS Number	Purity (%)
1	1-Ethyl-3-methylimidazolium bis(trifluoromethanesulfonyl)imide	[EMIm][TFSI]	174899-82-2	99.9
2	1-Butyl-1-methylpyrrolidinium bis(fluorosulfonyl)imide	[Pyr $_{1,4}$][FSI]	1057745-51-3	99.9
3	N-Propyl-N-methylpyrrolidinium bis(trifluoromethanesulfonyl)imide,	[Pyr $_{1,3}$][TFSI]	852620-97-4	99.9
4	N-Propyl-N-methylpyrrolidinium bis(fluorosulfonyl)imide	[Pyr $_{1,3}$][FSI]	852620-97-4	99.9
5	1-Butyl-1-methylpyrrolidiniumbis (trifluoromethanesulfonyl)imide	[Pyr $_{1,4}$][TFSI]	1057745-51-3	99.9
6	1-Butyl-1-methylpyrrolidinium bis(fluorosulfonyl)imide	[Pyr $_{1,4}$][FSI]	1057745-51-3	99.9
7	N-Pentyl-N-methylpyrrolidinium bis(trifluoromethanesulfonyl)imide	[Pyr $_{1,5}$][TFSI]	-	99.9
8	1-Methyl-1-(2-methoxyethyl)pyrrolidinium Bis(trifluoromethanesulfonyl)imide	[Pyr $_{1,102}$][TFSI]	-	99.9
9	1-Methyl-1-(2-methoxyethyl)pyrrolidinium Bis(fluorosulfonyl)imide,	[Pyr $_{1,102}$][FSI]	-	99.9
10	1-Methyl-1-(2-methoxypropyl)pyrrolidinium Bis(trifluoromethanesulfonyl)imide	[Pyr $_{1,103}$][TFSI]	-	99.9
11	N-butyl-N-methylpiperidinium bis(trifluoromethanesulfonyl)imide	[Pip $_{1,4}$][TFSI]		99.9
12	1-Methyl-1-propylpiperidinium bis(trifluoromethanesulfonyl)imide	[Pip$_{1,3}$][TFSI]	608140-12-1	99.9
13	1-Methyl-1-propylpiperidinium bis(fluorosulfonyl)imide	[Pip$_{1,3}$][FSI]	911303-46-3	99.9
14	N-Trimethyl-N-propylammonium bis(fluorosulfonyl)imide	[N $_{1,1,1,3}$][FSI]	-	99.9
15	N-Trimethyl-N-butylammonium bis(trifluoromethanesulfonyl)imide	[N $_{1,1,1,4}$][TFSI]	258273-75-5	99.9
16	N-Trimethyl-N-hexylammonium bis(trifluoromethanesulfonyl)imide	[N $_{1,1,1,6}$][TFSI]	210230-43-6	99.9
17	N-Tributyl-N-methylammonium bis(trifluoromethanesulfonyl)imide,	[N $_{4,4,4,1}$][TFSI]	405514-94-5	99.9
18	N-ethyl-N,N-dimethyl-N(2methoxyethyl)ammonium bis(trifluoromethylsulfonyl)imide	[N $_{1,1, 2, 102}$][TFSI]	557788-37-1	99.9
19	N-ethyl-N,N-dimethyl-N(2methoxyethyl)ammonium bis(fluorosulfonyl)imide	[N $_{1,1, 2, 102}$][FSI]	1235234-35-1	99.9
20	N,N-diethyl-N-methyl-N-(2-methoxyethyl)ammonium bis(trifluoromethanesulfonyl)imide	[N $_{2,2,1, 102}$][FSI]	557788-37-1	99.9
21	N,N-Dimethyl-N-Ethyl-N-Phenyl ethylammonium Bis(trifluoromethanesulfonyl)imide	[N$_{1,1,2}$, PhenylEth][TFSI]	-	99.9
22	N,N-Dimethyl-N-ethyl-N-benzylAmmonium Bis(trifluoromethanesulfonyl)imide	[N $_{1,1,2}$,Benz][TFSI]	-	99.9

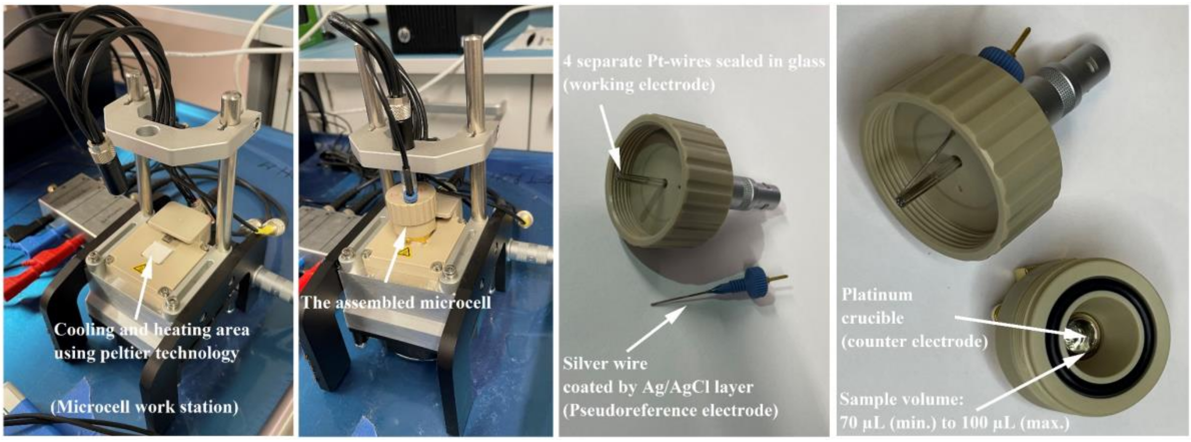

Figure S1: Microcell assembly and temperature control stand used in the study.

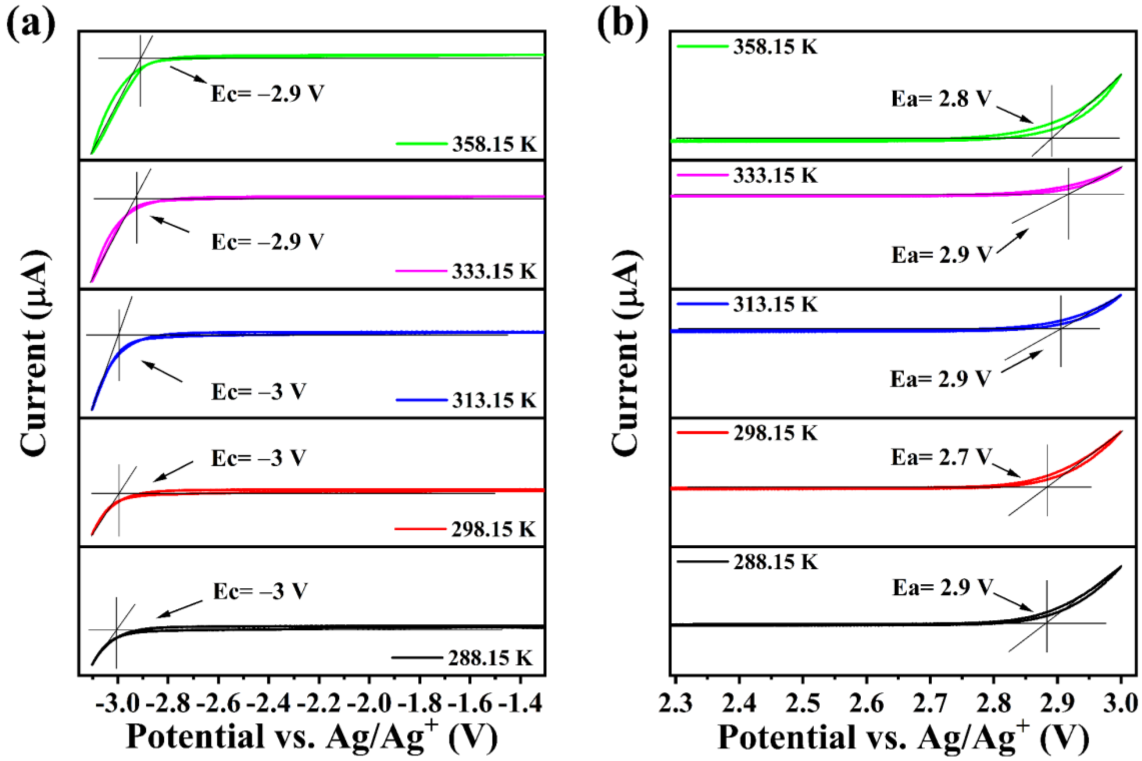

Figure S2: Method of using tangents to estimate potential limits (a) For E_a and (b) For E_c. Sample CV shown for [N $_{1,1,1,4}$][TFSI].

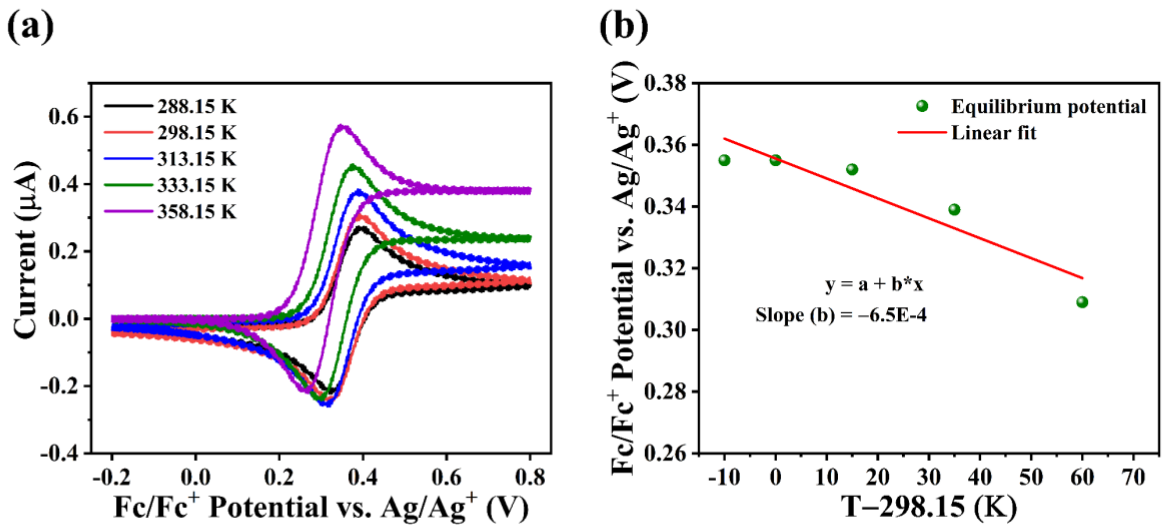

Figure S3: Calibration of the Ag/Ag^+ quasi reference vs. Fc/Fc^+ using the cell setup. (a) CV of 0.1 mM Fc/Fc^+ in [EMim][TFSI] at 50 mV/s, at various test temperatures (b) Linear fit of Fc/Fc^+ equilibrium potential vs. Ag/Ag^+ at different deviations from standard Temperature, obtained from the CVs in (a).

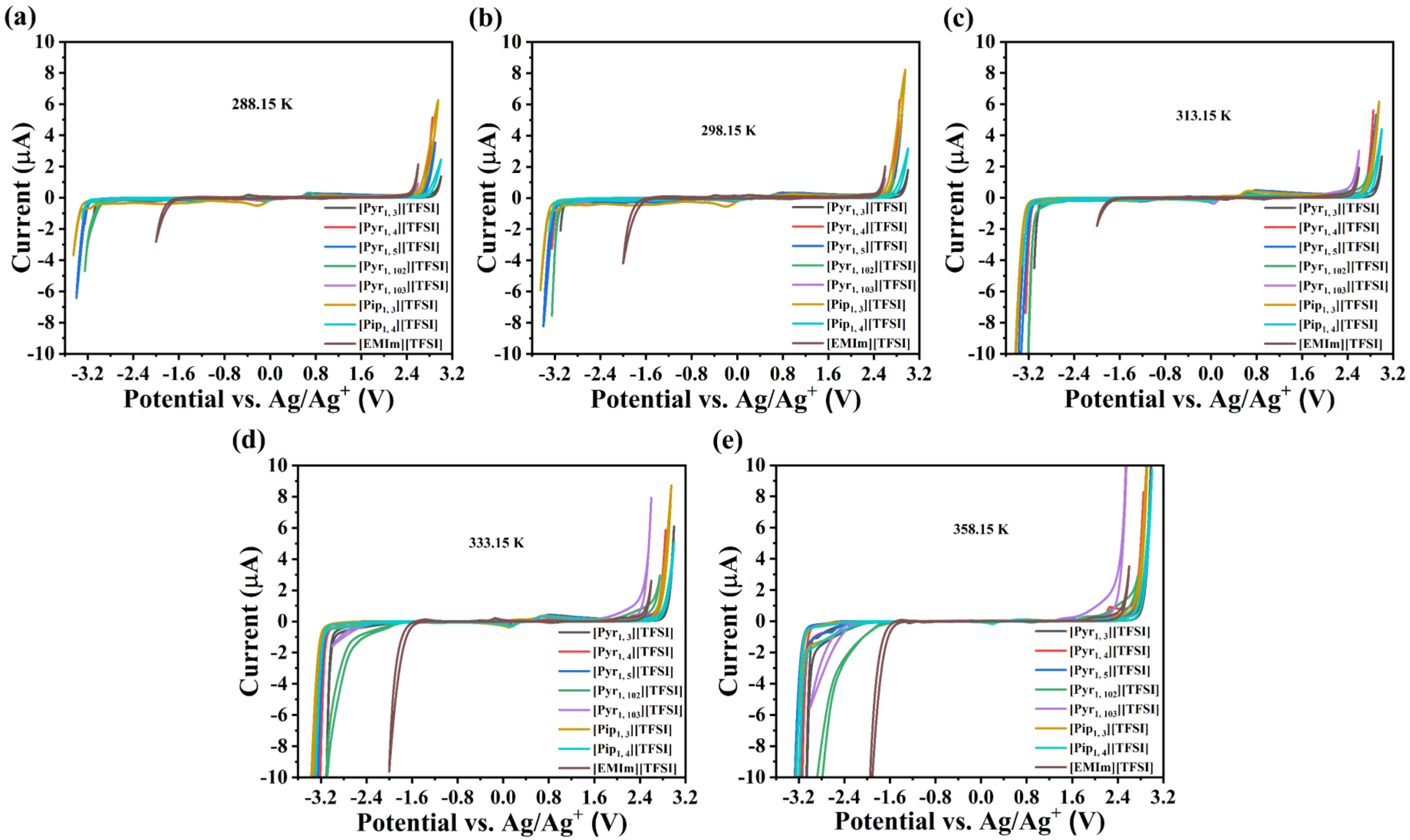

Figure S4: CVs of ILs with pyrrolidinium, piperidinium and imidazolium cations in combination with [TFSI] anion at; (a) 288.15 K. (b) 298.15 K. (c) 313.15 K. (d) 333.15 K. and (e) 358.15 K.

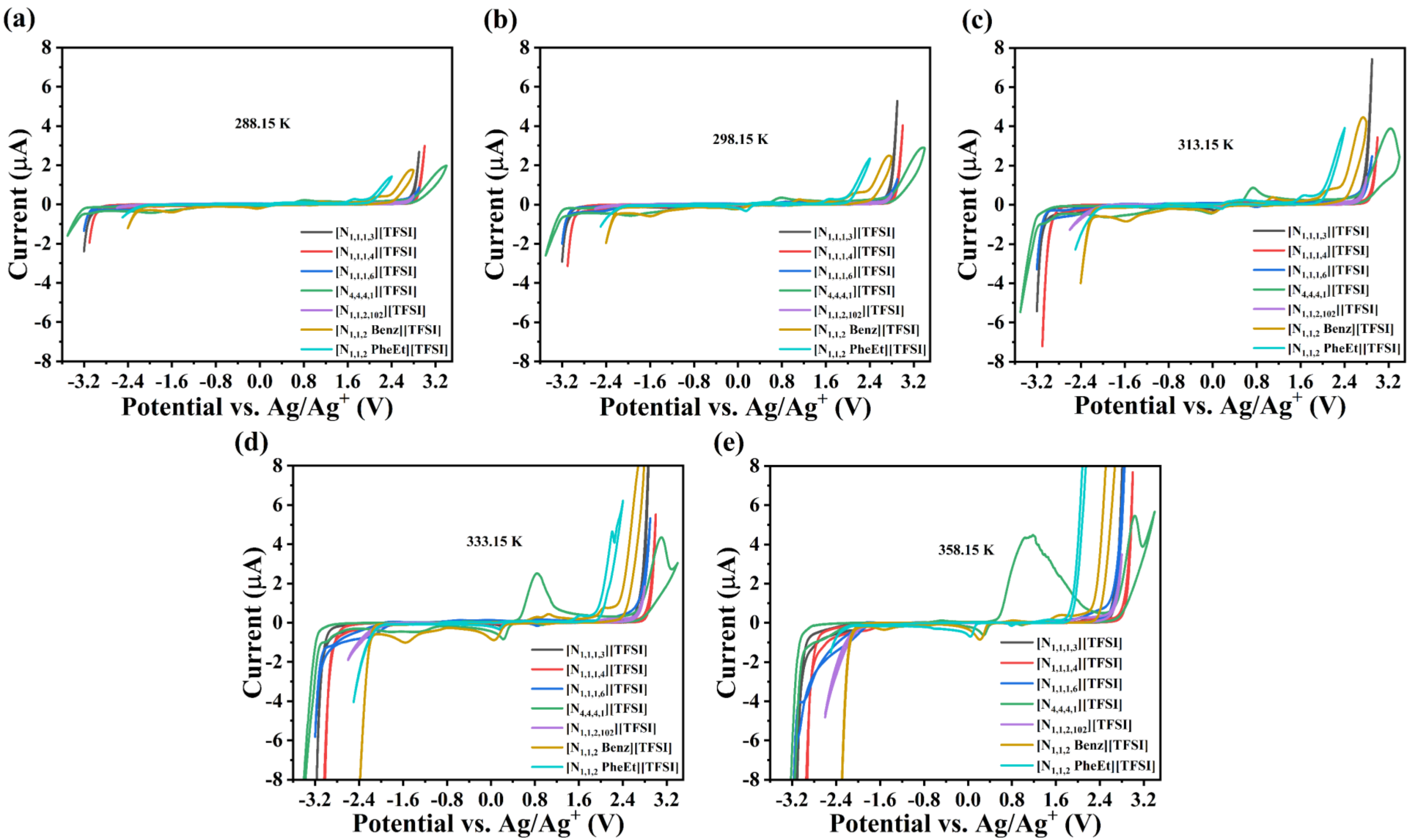

Figure S5: CV of ILs with ammonium cations in combination with [TFSI] anion at; (a) 288.15 K. (b) 298.15 K. (c) 313.15 K. (d) 333.15 K. and (e) 358.15 K.

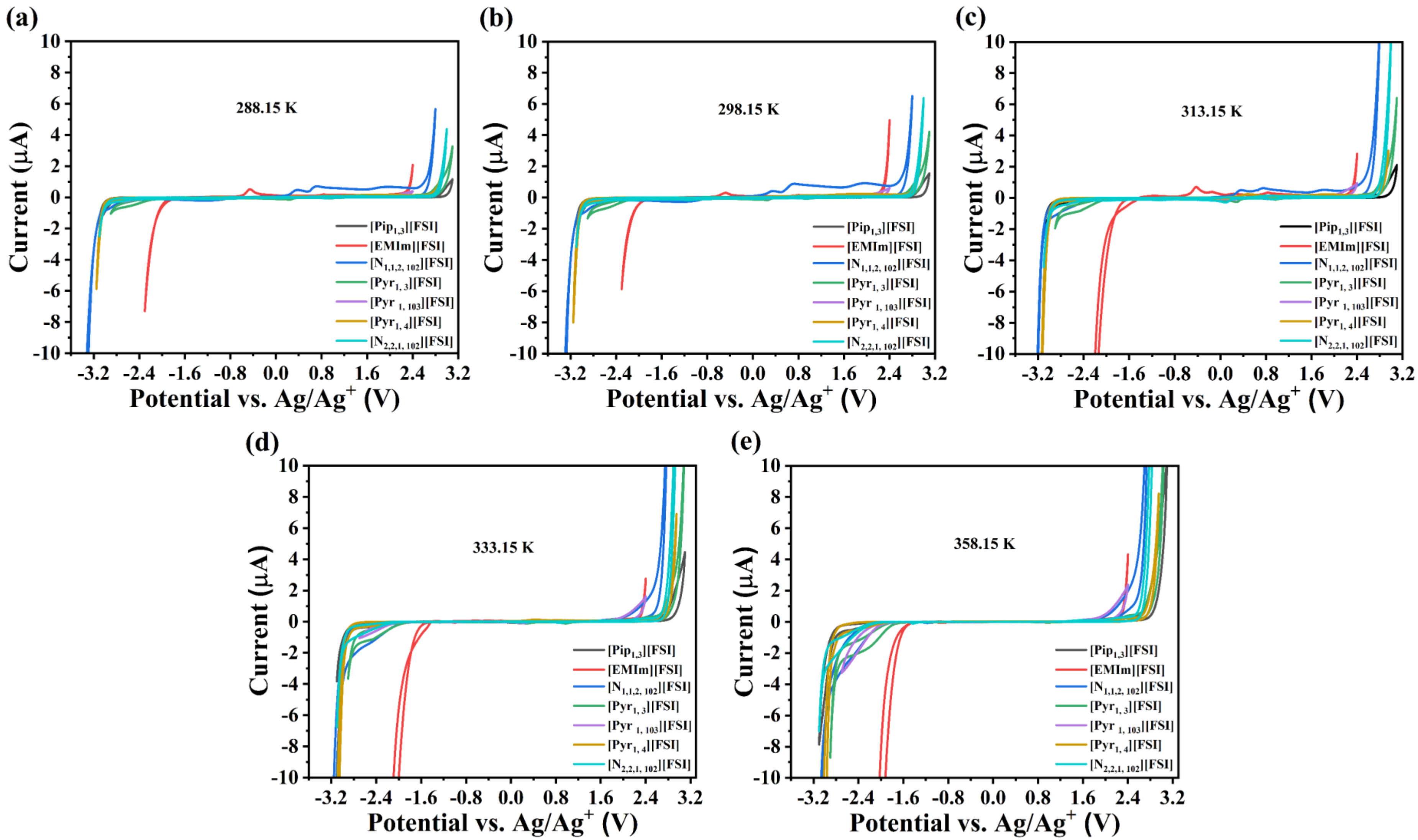

Figure S6: CV of ILs with pyrrolidinium, piperidinium and imidazolium cations in combination with [FSI] anion at; (a) 288.15 K. (b) 298.15 K. (c) 313.15 K. (d) 333.15 K. and (e) 358.15 K.
